# Non-Productive Infection of Glial Cells with SARS-CoV-2 in Hamster Organotypic Cerebellar Slice Cultures

**DOI:** 10.3390/v14061218

**Published:** 2022-06-03

**Authors:** Lise Lamoureux, Babu Sajesh, Jessy A. Slota, Sarah J. Medina, Matthew Mayor, Kathy L. Frost, Bryce Warner, Kathy Manguiat, Heidi Wood, Darwyn Kobasa, Stephanie A. Booth

**Affiliations:** 1 One Health Division, Public Health Agency of Canada, National Microbiology Laboratory, 1015 Arlington St., Winnipeg, MB R3E 3R2, Canada; lise.lamoureux@phac-aspc.gc.ca (L.L.); babu.sajesh@phac-aspc.gc.ca (B.S.); jessy.slota@phac-aspc.gc.ca (J.A.S.); sarah.medina@phac-aspc.gc.ca (S.J.M.); matthew.mayor@phac-aspc.gc.ca (M.M.); kathy.frost@phac-aspc.gc.ca (K.L.F.); kathy.manguiat@phac-aspc.gc.ca (K.M.); heidi.wood@phac-aspc.gc.ca (H.W.); 2 Department of Medical Microbiology and Infectious Diseases, Faculty of Health Sciences, University of Manitoba, 730 William Ave., Winnipeg, MB R3E 0W3, Canada; darwyn.kobasa@phac-aspc.gc.ca; 3 Special Pathogens, Public Health Agency of Canada, National Microbiology Laboratory, 1015 Arlington St., Winnipeg, MB R3E 3R2, Canada; bryce.warner@phac-aspc.gc.ca

**Keywords:** SARS-CoV-2, COVID-19, brain, organotypic culture, astrocytes, microglia, neuroinflammation

## Abstract

The numerous neurological syndromes associated with COVID-19 implicate an effect of viral pathogenesis on neuronal function, yet reports of direct SARS-CoV-2 infection in the brain are conflicting. We used a well-established organotypic brain slice culture to determine the permissivity of hamster brain tissues to SARS-CoV-2 infection. We found levels of live virus waned after inoculation and observed no evidence of cell-to-cell spread, indicating that SARS-CoV-2 infection was non-productive. Nonetheless, we identified a small number of infected cells with glial phenotypes; however, no evidence of viral infection or replication was observed in neurons. Our data corroborate several clinical studies that have assessed patients with COVID-19 and their association with neurological involvement.

## 1. Introduction

SARS-CoV-2 infection primarily causes a respiratory disease that can be severe or fatal. Concerning neurological problems such as anosmia (the loss of smell), dysgeusia (the loss of taste), and headaches have been reported in up to half of all COVID-19 patients during acute disease and can linger during recovery [1,2]. Long-term effects are reported in up to a third [3,4,5] of all patients, including psychiatric malaise, debilitating fatigue, and trouble thinking clearly or “brain fog”. In severely ill patients, consequences of infection have included stroke, intracranial haemorrhage and onset or worsening of dementia [6,7].

It remains an open question whether the neurological symptoms of SARS-CoV-2 are caused by direct infection of brain cells, or by the impact of extensive peripheral inflammation or autoimmunity [8,9]. Critically ill patients suffer a ‘cytokine storm’, whereby proinflammatory cytokines can cross a compromised blood brain barrier (BBB), leading to bleeding in the brain and activation of perivascular microglia and astrocytes [10]. Other secondary effects include hypoxia, hypercoagulability, and multisystem organ failure [11]. Studies have detected evidence of neuroinvasion, viral infiltration, and replication in the brain of a very small number of patients [8,12,13,14]. However, the majority of recent reports have failed to detect SARS-CoV-2 proteins or viral RNA in the brain tissue of patients who have succumbed to COVID-19, whether or not neurological symptoms were present [11,15,16].

Several animal models of SARS-CoV-2 infection have been established including hamsters, ferrets, and non-human primates [17]. These models recapitulate SARS-CoV-2 induced pathology, viremia and lung tissue tropism, but do not provide evidence of SARS-CoV-2 invasion into brain tissue [17]. In these models, infection with SARS-CoV-2 is dependent on the expression of ACE2, the SARS-CoV-2 receptor [18,19,20,21]. The Syrian hamster is well-established for the study of SARS-CoV-2, as it closely models human disease. Respiratory infection and lung pathology peak 4-6 days following exposure, and animals exhibit weight loss, mild lethargy, ruffled fur, and completely recover by 14 days [22,23,24,25]. No overt neurological signs were exhibited by the animals and no pathology or viral proteins were identified in brain tissues. However, this might be due to mild pathology and lack of BBB breakdown seen in the wild-type hamster model.

Organotypic brain slice cultures are a well-established technique most frequently used to study neurological conditions such as neurodegeneration. The use of organotypic slice cultures circumvents the need for neuroinvasion because they lack an intact vascular system and effective BBB. Several neurotropic infectious diseases have been modeled in brain organotypic cultures, in particular HSV-1 and Zika virus, that can both cause severe neurological disease [22,23]. As these slice cultures maintain a 3D organisation while preserving cytoarchitecture and cell populations, they are an accessible system for studying neurotropism and identifying permissive cell types.

In this study, we used cerebellar organotypic slice cultures to examine SARS-CoV-2 infection of hamster brain tissue in the absence of a BBB. Viral RNA was detected via PCR for up to 2 weeks post infection, although waning levels of live virus indicated non-productive replication. By indirect immunofluorescence imaging assays, we observed evidence of SARS-CoV-2 infection in astrocytes and microglia. RNAseq data suggest a small number of transcripts were altered in SARS-CoV-2 inoculated cerebellar sections. Overall, our data indicates that a small population of glial cells may be permissive for SARS-CoV-2; however, productive infection and replication in hamster brain slices did not occur.

## 2. Materials and Methods

### 2.1. Cell Lines and Virus

Vero-e6 cells (CRL-1586 ATCC) were maintained in high-glucose Eagle’s Minimum Essential Medium (MEM) supplemented with L-glutamine and 10% heat-inactivated foetal bovine serum (Fisher Scientific, Waltham, MA, USA). SARS-CoV-2 (hCoV-19/Canada/ON-ON-VIDO-01-2/2020, EPI_ISL_425177) inoculum was produced in Vero-e6 cells as described previously [26]. It was titered at 1370 plaque forming units (PFU) per µL using a previously described plaque assay [27].

### 2.2. Animal Experiment

Golden Syrian hamsters were purchased from Charles River Laboratories (Wilmington, MA, USA) and challenged with 10^5^ TCID_50_ of the virus in 100 µL serum-free Dulbecco’s Modified Eagle Medium–High Glucose (DMEM) administered through the intranasal route (50 µL per nostril). On day 5 post challenge, animals were euthanized and brains removed. All the procedures were approved by the Animal Care Committee of the Canadian Science Centre for Human and Animal Health and followed the guidelines of the Canadian Council for Animal Care.

### 2.3. Immunohistochemistry

Brain samples were fixed in 10% neutral buffered formalin, trimmed, put into cassettes and processed using the HistoCore Pearl Tissue Processor (Leica, Wetzlar, Germany). Using the HistoCore Arcadia embedding station (Leica) paraffin-embedded blocks were produced and 5-micron-thick coronal serial sections of the mid-brain were cut on a microtome (HistoCore AutoCut, Leica). The slices were mounted on positively charged slides and baked overnight at 37 °C, manually deparaffinized and rehydrated using two 3 min xylene incubations and then two 2 min 100% ethanol incubations, two 2 min 95% ethanol incubations and then distilled H_2_O for 2 min. Endogenous enzyme activity was blocked by immersing the sections in 3% hydrogen peroxide for 10 min and rinsed with distilled H_2_O. Subsequent antigen retrieval was done by placing the slides in 10 mM sodium citrate buffer (pH 6.0) and incubating in BioCare’s decloaking chamber using their 110 °C antigen retrieval protocol. A 1:2000 dilution of Iba1 primary antibody (019-19741, Wako) was added to the sections and incubated overnight in a humidity chamber at 4 °C. Antibody signal was detected using the Polink-2 Plus HRP Rabbit DAB Detection System (Golden Bridge International GBI, Inc., Bothell, WA, USA) following the manufacturer’s recommendation. Sections were counterstained with Hematoxylin 560 MX (Leica) for 2 min, rinsed and decolorized with Define MX-AQ (Leica), blued with Blue Buffer 8 (Leica), then dehydrated, cleared, and mounted using Permount (Fisher Scientific). Slides were scanned using the AxioScan Microscope slide scanner (Zeiss, Oberkochen, Germany).

### 2.4. Hamster Brain Organotypic Culture Preparation

Brain tissue was collected from golden Syrian hamster pups at post-natal day 9–11 and placed in ice-cold Geys balanced salt solution supplemented with 1 mM kynurenic acid and 0.6 mM glucose (GBSSK buffer). The cerebellum was dissected from the brain, immersed in 2% low-melting point agarose (37 °C) and solidified on ice. Sagittal sections (300 μm in thickness) were prepared from the cerebellar tissue using a vibratome (Microm HM650V, Thermo Fisher Scientific) and transferred to Millicell CM transwell inserts within 6-well culture plates containing ice cold GBSSK. Once all slices were transferred, GBSSK was replaced with 1 mL of warm slice culture medium (SCM; MEM supplemented with 20% horse serum, 1 mg/L insulin, 5.2 mM NaHCO3 and 30 mM HEPES) and maintained at 37 °C in a humidified incubator supplemented with 5% CO_2_. Medium was changed thrice every week.

### 2.5. SARS-CoV-2 Infection and TCID_50_

Hamster cerebellar brain slices were treated with either 0 (Mock), 450 or 6850 PFU of SARS-CoV-2 by pipetting 2 µg of diluted inoculum directly onto each slice. Media and/or lysate was collected from SARS-CoV-2 treated or Mock treated organotypic slice cultures at various time points (i.e., 1, 2, 3, 5, 7 and 21 days post infection; DPI).

Media collected from organotypic cultures was assayed for live virus using a 50% tissue culture infective dose (TCID_50_) assay. Briefly, Vero-e6 cells were cultured to confluence in 96-well plates. Samples were serial diluted 10-fold in DMEM supplemented with 2% FBS and Vero-e6 cells were treated in quadruplicate with each dilution in a final volume of 150 µL. Vero-e6 cells were maintained at 37 °C for 96 h post infection, at which point cells were monitored for cytopathic effect and TCID_50_ values were calculated using the Spearman-Karber method [28,29].

### 2.6. RNA Isolation and Quantitative RT-PCR

Viral RNA was isolated from organotypic cultures using the QIAamp viral RNA extraction kit (Qiagen, Verlo, The Netherlands) following manufacturer’s instructions and quantified using a Nanodrop™ (ThermoFisher Scientific). Total RNA from organotypic slices were isolated using the RNeasy mini kit (Qiagen) and quantified using the nanodrop. cDNA was generated using the high-capacity cDNA reverse transcriptase (ThermoFisher Scientific). Real-time PCR was carried out using the Taqpath reagent (ThermoFisher Scientific) and the primers/probes recommended by the US CDC SARS-CoV-2 RUO qPCR Primer & Probe Kit for N1 (catalog #10006713, Integrated DNA Technologies) with 2019-nCoV_N_Positive Control (catalog #10006625, Integrated DNA Technologies).

### 2.7. Illumina Next-Generation Sequencing

Next-generation sequencing libraries were prepared from 100 ng of total RNA using the TruSeq Stranded Total RNA Library Prep Gold (Illumina, San Diego, CA, USA) with the TruSeq RNA Single Indexes Set B (Illumina). The quality of library preparations was checked on tapestation instrument (Agilent, Santa Clara, CA, USA) using the high-sensitivity D5000 screentape (Agilent). Libraries were sequenced to a depth of 20–30 million raw reads (75 PE) with a NextSeq 550 instrument using high-output flow cells (150 cycles, Illumina).

### 2.8. Indirect Immunofluorescence

Following inoculation with SARS-CoV-2 or mock treatment, brain slices were fixed with 4% paraformaldehyde (overnight) either 1 h post infection (0.042 DPI) or 1, 2, 3, 5, 6, 7, 14, 15, and 16 DPI and were used for subsequent assays listed below. A previously described immunofluorescence assay [30,31] (with minor modifications) was used to image whole-mount cerebellar sections. Briefly, brain slices (N = 4 per time point) were permeabilized with 1% Triton-X100 in phosphate-buffered saline (PBS; pH 7.4) (FischerScientific) for 1 h, washed twice with PBS, and incubated with primary antibodies (Supplementary Table S1) overnight at 4 °C. Samples were washed twice with PBS, once with 0.1% Triton-X100 in PBS and twice again with PBS and incubated with secondary antibodies for 1 h at room temperature. Following washes as described above, nuclei were counterstained with DAPI and confocal images were acquired on a Zeiss LSM700 microscope. Images were saved as greyscale TIFF files for individual channels and panels were generated on GNU image manipulation program (GIMP) 2.10.30. The assays were repeated at least one additional time. Unless specified, SARS-CoV-2 nucleocapsid was visualized using AlexaFluor-488 conjugated secondary antibody and was detected as green fluorescence (signal). Total number of infected cells was counted for each slice and the maximal continuous distance of signal from the centre of the infected cell was measured using image analysis software Zen Version 3.4 (Zeiss). For infected cell counts and maximal distance; N = 4 for each time point, Student’s *t*-test (unpaired, two-tailed) were performed, and graphs are presented as mean ± SEM unless otherwise specified. Semi-quantitative analysis was performed using Fiji (Image J) as described in Supplemental Methods.

### 2.9. In Situ Hybridization Assays

Slices were embedded in optimal cutting temperature compound (O.C.T.; TissueTek^®^, Torrance, CA, USA), and 15 µm sections were cut on a CM1860 cryostat (Leica Biosystems). Each section was processed for in situ hybridization (ISH) following the manufacturer’s recommended protocol. Probes to detect either *Map2* (Cat# 313211), *Gfap* (Cat# 431151), or + sense (Cat# 859151-C2) and −sense (Cat# 848561) of SARS-CoV-2 RNA strands were purchased from Advanced Cell Diagnostics (RNAScope^®^, Hayward, CA, USA). ISH was performed on brain slices (N = 4 per time point) using a fluorescent multiplex reagent kit to simultaneously detect SARS-CoV-2 and either *Map2* or *Gfap*. To detect microglia, we used an antibody to detect *Iba1* by immunocytochemistry following ISH to detect SARS-CoV-2. Nuclei were counterstained with DAPI and confocal images were acquired, image panels were generated as described above. The assay was repeated one additional time.

### 2.10. Pre-Processing of RNASeq Reads

Raw fastq files were cleaned by removing low-quality sequences with Trimmomatic and removing ribosomal RNA reads with Bowtie2. The cleaned reads were then mapped to the (golden Syrian) hamster genome using HISAT2, and transcripts were counted using FeatureCounts. FastQC was used to assess the quality of the sequencing reads before cleaning, and after alignment to the host reference genome. Reads that failed to map to the host genome were then mapped to the SARS-CoV-2 genome using HISAT2 and viral reads were counted using FeatureCounts. The host and viral read count files were merged into a single matrix file for further processing using a custom R script.

### 2.11. Normalization and Differential Gene Expression Analysis

The raw read count matrices were normalized in R using DESeq2 [32]. For measuring host gene expression, the host read count matrix was supplied individually, while for measuring viral gene expression, the merged matrix of host + viral reads was used. DESeq2 was used for differential expression analysis by comparing SARS-CoV-2 treated vs. mock-treated slice culture samples at 1, 5 and 14 DPI. Differentially expressed genes were defined as having baseMean > 15, Log2 fold change magnitude > 0.5 and Bonferroni-Hochberg corrected *p*-value < 0.1. We used these relaxed criteria for identifying differentially expressed genes between SARS-CoV-2 and mock-treated samples because very few differentially expressed genes were identified according to standard criteria (e.g., *p*-value < 0.05). For comparing transcriptional profiles between timepoints, differential expression analysis was conducted with DESeq2 between samples at 5 vs. 1 DPI and 14 vs. 1 DPI. In this case, differentially expressed genes were identified using criteria of baseMean > 15, absolute value of Log2 fold change > 0.85 and Bonferroni–Hochberg-corrected *p*-value < 0.05.

### 2.12. Cell-Type Annotation of Genes

Differentially expressed genes were annotated based on a list of known cell-type-enriched transcripts [33]. From this list, each transcript was assigned to one of 6 broadly defined brain cell types—astrocytes, endothelial cells, microglia, neurons, oligodendrocyte, oligodendrocyte progenitor cells (OPCs), or unassigned.

### 2.13. Gene Set Enrichment Analysis

Functional enrichment was performed on lists of differentially expressed genes that were either increased (up) or decreased (down) in abundance at each comparison (SARS vs. Mock at 1, 5 and 14 DPI, in addition to all samples at 5 vs. 1 DPI and 14 vs. 1 DPI). Each list was supplied to Enrichr [34] (using the enrichR R-package) and functional enrichment was performed against the following databases of gene sets: BioPlanet 2019, WikiPathways 2019 Human, GO Biological Process 2021, GO Molecular Function 2021, and GO Cellular Component 2021. Enriched gene sets were ranked by the *p*-value supplied by enrichR, and the top gene sets were selected for visualization.

### 2.14. Data Visualization

All plots were prepared in R using ggplot2, except heatmaps that were prepared using pheatmap. Principle component analysis (PCA) was performed with the normalized log2-transformed host read matrix using DESeq2. Hierarchical clustering was performed with pheatmap by supplying the matrix of z-scores calculated from the normalized log2-transformed read counts (z-score = (x-mean(x))/stddev(x)). Enrichment plots were prepared from the output of Enrichr by plotting the –log10(*p*-value) against each term and mapping the corresponding ‘combined score’ value to colour.

## 3. Results

### 3.1. Non-Productive Infection with SARS-CoV-2 in Organotypic Slice Cultures of Hamster Brain Tissue

**Figure 1:**
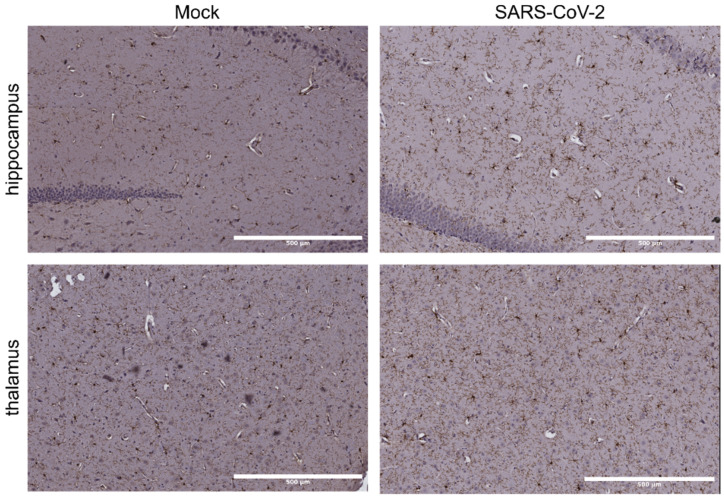
Mild increase in IBA1 staining in the hippocampus and thalamus of SARS-CoV-2-infected hamsters. IHCwith IBA1 and counterstained with hemotoxylin was performed in the hippocampus (top row) and thalamus (bottom row) of brain sections taken from hamsters that were either mock-infected (left column) or infected with SARS-CoV-2 (right column). Microglia in infected tissue, particularly the hippocampal region, show changes in phenotype such as thickening and increased staining of processes, and an increase in staining of the soma of some cells. Microglia remain in a ramified state; activated microglia with amoeboid phenotypes were not observed. Scale bar indicates 500 µm.

We performed immunohistochemistry on formalin-fixed brains of hamsters infected with SARS-CoV-2 that showed clinical signs of disease and lung pathology at 5-DPI. No difference in brain pathology was observed between infected and mock-infected hamsters, and no staining was observed using an antibody to SARS-CoV-2 nucleocapsid protein (Supplementary Figure S1). However, we saw some increase in staining with the antibody IBA1 that binds microglia and morphological changes such as shortening and thickening of some glial processes (Figure 1). Microglia were not in a fully activated state that would be characterised by an amoeboid shape, and may indicate a mild transient response. To determine whether this is due to an inability of the virus to cross the blood brain barrier we used an organotypic culture system to investigate the permissivity of cells of the central nervous system infection with SARS-CoV-2.

**Figure 2:**
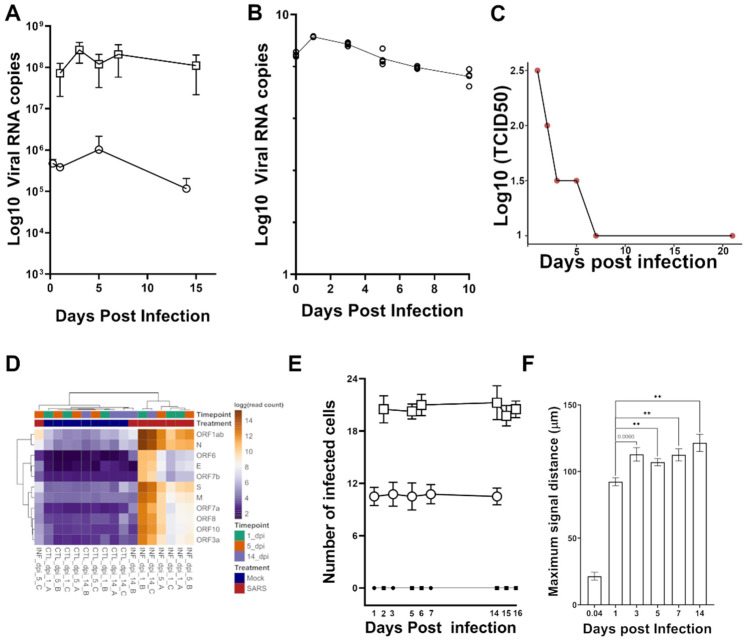
SARS-CoV-2 shows negligible infectivity in hamster brain cerebellar slice culture. Viral RNA in (**A**) lysate or (**B**) culture medium from cerebellar slice cultures infected with SARS-CoV-2 was quantified using qRT-PCR targeting the nucleocapsid sequence. Either 450 (**A**; open circles) or 6850 (**A**; open squares) PFU of SARS-CoV-2 inoculum was used to challenge the cerebellar slice cultures. DPI is indicated on the *x-*axes and the viral RNA (log_10_) quantified by qRT-PCR is indicated on the *y*-axes. Live virus in hamster brain cerebellar slice cultures was quantified (**C**) following inoculation with SARS-CoV-2 using TCID_50_ assay. DPI are indicated on the *x-*axis and the viral titer is indicated on the *y*-axis. (**D**) Hierarchical clustering of RNAseq raw read counts mapping to SARS-CoV-2 transcripts were normalized and abundance was calculated as log2 transformed read counts using DESeq2. Bar diagrams depicting (**E**) the number of cells infected with SARS-CoV-2; circles and squares indicate slices infected (white) with 450 PFU and 6850 PFU respectively as compared to relevant controls (black) and (**F**) maximum signal distance (in µm) of viral nucleocapsid protein from the centre of the infected cells as visualized by indirect immune fluorescence imaging. The time points are indicated on the *x*-axis; 0.04 is 1 h post infection. Values are depicted as mean ± SEM; N = 4 ** indicates a statistical significance with a *p*-value < 0.005; actual *p*-value (0.006) is indicated otherwise.

Cerebellar brain slices were cut using a vibratome and kept in culture for 1 week prior to challenge with virus at two different concentrations. The presence of viral RNA in cell lysate was detected at various time points over two weeks following challenge with either 450 or 6850 PFU (Figure 2A). Evidence of viral genome replication was only apparent at day 1, followed by a gradual decline over time. Similarly, viral RNA was detected in culture media at 1-DPI, followed by a decline over time (Figure 2B). Virus was detected by TCID_50_ up to 7-DPI (Figure 2C). In addition, we were able to detect reads mapping to the SARS-CoV-2 genome using RNAseq at 1-, 5- and 14- DPI. (Figure 2D). Viral reads were absent in one of the samples collected at 5 and one at 14 DPI, consistent with the waning levels of RNA and virus detected by RT-PCR and TCID_50_ data. We concluded that a small population of cells in hamster brain are permissive to infection, but that this is non-productive.

To determine the extent of infection by SARS-CoV-2, we used IF to count the total number of cells in each cerebellar section that stained positive for the viral nucleocapsid protein. We found that the number of nucleocapsid^+^ cells per slice correlated with the titer of viral inoculum used to treat the tissue—an average of 10 nucleocapsid^+^ cells were seen in slices treated with 450 PFU while ~20 nucleocapsid^+^ cells were seen in those treated with 6850 PFU. The number of nucleocapsid^+^ cells were similar at each time point from 1 DPI onwards (Figure 2E), and student’s *t*-tests indicated no statistical significance in the number of nucleocapsid^+^ cells across time points. To determine if we were detecting residual virus inoculum, we also performed IF at 1 h following infection with virus (data not shown). In this case, less than 1 nucleocapsid^+^ cell per slice was detected. We were therefore confident that those cells labelled 24 h or more post infection contained newly translated viral proteins.

The consistent number of nucleocapsid^+^ cells detected across timepoints indicates that SARS-CoV-2 did not spread from cell to cell. As an additional surrogate measure for spread of infection, we measured the maximal distance of continuous nucleocapsid signal from the centre of each infected cell (Figure 2F and Supplementary Figure S2). Continuous nucleocapsid fluorescence signal was detected up to 21.5 µm away from the centre of the cell on average after 1 h post infection. Subsequently, this distance was an average of 92.33 µm, 112.83 µm, 107 µm, 112.5 µm, and 121.5 µm on 1, 3, 5, 7, 14 DPI, respectively. There was no statistical significance between time points, except for 1 DPI, as observed by a two-tailed unpaired *t*-test. A *p*-value < 0.005 was observed when maximal signal distances were compared between 1-DPI with 5- (*p*-value = 0.0046), 7- (*p*-value = 0.0038) and 14-(*p*-value = 0.0022) DPI. Collectively, we failed to observe evidence of cell-to-cell spread of SARS-CoV-2.

### 3.2. SARS-CoV-2 Infects Glia but Not Neurons in Infected Hamster Cerebellar Slice Cultures

**Figure 3:**
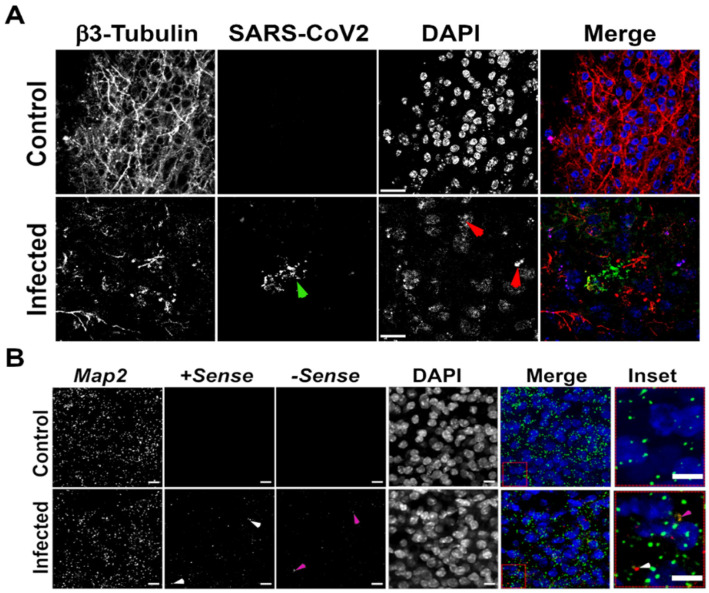
SARS-CoV-2 does not infect neurons in a hamster brain cerebellar slice culture. Representative images of hamster brain cerebellar slices labelled to visualize (**A**) β3-tubulin (red) and SARS-CoV-2 nucleocapsid (green) by indirect immunofluorescence or (**B**) neuronal (*Map2*; green) and SARS-CoV-2 markers by RNA ISH. Nuclei were counterstained with DAPI (blue). Merge is a composite of all individual images. Arrowheads identify either viral nucleocapsid protein (green; (**A**)), +sense (white; (**B**)) or -sense (magenta; (**B**)) viral RNA strands indicating areas of active infection and replication respectively. The red arrowheads (**A**) indicate fragmenting nuclei and blebbing, surrogate markers for apoptosis. Scale bar = 10 βm.

We were interested to determine whether the small population of nucleocapsid^+^ cells belonged to a particular cell-type. We co-stained with antibodies that recognize specific cell markers of neurons (β3-Tubulin) or glia (GFAP and IBA1). There was no overlap between β3-tubulin^+^ neurons and SARS-CoV-2 nucleocapsid signal (Figure 3A), suggesting neurons were not infected. However, we observed that β3-tubulin labelled cells in close proximity to SARS-CoV-2 labelled cells showed significant disruption of dendritic processes (green arrowhead; Figure 3A). In addition, these cells showed distinct phenotypic signs of apoptosis, including nuclear fragmentation and nuclear condensation (red arrowheads; Figure 3A). It was unclear whether this was related to SARS-CoV-2 infection, or the presence of inflammatory and/or cell death signalling molecules that could have been present in the inoculum. We further labelled the slices with a second neuron marker, MAP2, in addition to ISH probes for both positive- and negative-sense viral RNA strands (Figure 3B). Signals from both +sense (Figure 3B inset, red; white arrowhead) and –sense (Figure 3B magenta arrowhead) viral RNA strands were spatially resolved from MAP2 (green; inset Figure 3B), confirming that SARS-CoV-2 did not infect hamster neurons in cerebellar brain slices.

**Figure 4:**
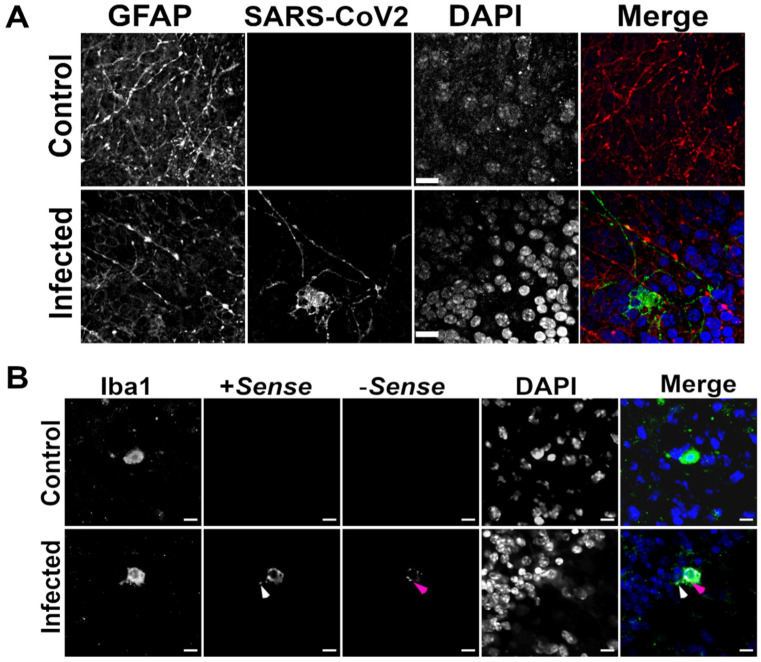
SARS-CoV-2 may infect glial cells in a hamster brain cerebellar slice culture. Representative images of hamster brain cerebellar slices identifying either astrocytic (Gfap (red); (**A**)) or microglial (Iba1 (green); (**B**)) markers and SARS-CoV-2 nucleopasid (green; (**A**)) or +sense (white arrowheads (red); (**B**)) or -sense (magenta arrowheads (yellow); (**B**)) viral RNA strands. Nuclei were counterstained with DAPI/ blue; merge depicts a composite image. Scale bar = 10 µm. We also calculated the corrected total intensity of each marker to compensate for background fluorescence (Supplementary Figure S4). Additionally, since both β3-Tubulin and Map2 were cytoskeletal markers, we used NeuN, a nuclear marker for neurons, to prevent unconscious observational bias of the surrogate markers we used. We found no overlap in fluorescence signals from probes identifying SARS-CoV-2 and NeuN. These data further confirm our observation that SARS-CoV-2 does not infect hamster neurons in organotypic cerebellar brain slices.

We next used an antibody to detect the astrocyte marker GFAP and in some instances saw a close association between cells labelled with GFAP and SARS-CoV-2 nucleocapsid. The morphology of these cells was reminiscent of ramified mature astrocytes (Figure 4A). GFAP and viral proteins were spatially associated (although did not overlap), and were often found to align as a single projection from the cell body. GFAP is an intermediate filament protein found in main processes but not the fine processes of astrocytes. This close association could either indicate viral proteins in processes that do not contain GFAP, or infection of a different cell type that are closely associated with astroglia. ISH of RNA probes targeting GFAP or +sense and −sense viral RNA strands coincided, providing some evidence SARS-CoV-2 can replicate within GFAP^+^ astrocytes (Supplementary Figure S3). In rare instances, infected cells did not share the phenotypic shape of astrocytes and were not proximate to GFAP staining. We posited that these cells could be microglia, and so we performed a combination of ISH using viral probes (as described above) and IF with an antibody against the microglia marker IBA1. We observed that these cells did indeed stain positive for IBA1 and for both +sense and −sense SARS-CoV-2 RNA (Figure 4B). Taken together, our data suggest that SARS-CoV-2 may infect GFAP^+^ and IBA1^+^ glial cells in hamster brain tissue, albeit inefficiently.

### 3.3. Transcriptional Differences Affiliated with Glia Were Detected in SARS-CoV-2-Treated Cerebellar Slice Cultures

**Figure 5:**
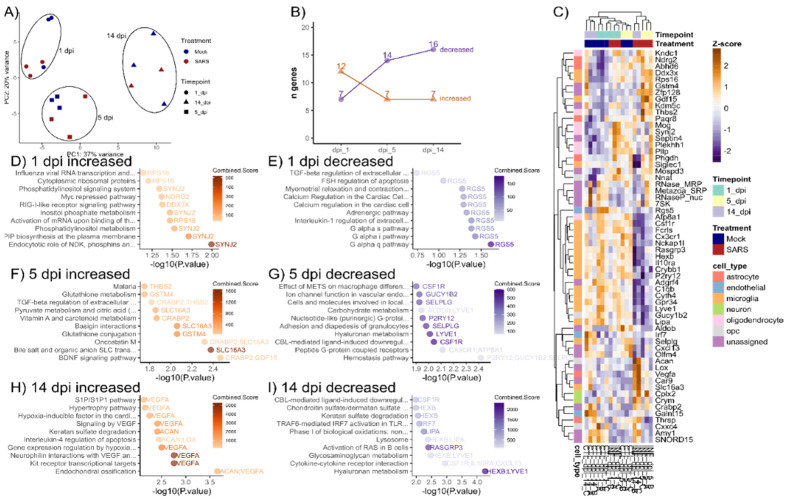
A limited transcriptional response is observed in hamster brain cerebellar slices infected with SARS-CoV-2. (**A**) PCA plot of all samples used for gene expression analysis at DPI 1, 5 and 14. (**B**) Number of differentially expressed genes in cerebellar sections at 1, 5 and 14 DPI. Differentially expressed genes were defined as mean read count >25, absolute value log2 (fold change) > 0.5 and FDR corrected *p*-value < 0.1. (**C**) Hierarchical clustering of differentially expressed genes at DPI 1, 5 and 14. Z-scores were calculated from log2 transformed normalized read counts and used for hierarchical clustering. (**D**–**I**) Enriched BioPlanet2019, WikiPathways 2021 and GO Biological Process 2021 pathways within list of differentially expressed genes. The combined list of differentially expressed genes was supplied to Enrichr for enrichment analysis.

We used RNAseq to characterize any transcriptional differences in the cerebellar slices following inoculation with SARS-CoV-2 at 1, 5 and 14 DPI. As expected, PCA showed that differences between the samples such as the time in culture were significant (Figure 5A and Supplementary Figure S5). However, we were able to identify some transcripts that were altered in abundance in association with SARS-CoV-2 infection at each time point. Using the criteria of a |log2 fold change| > 0.5 and FDR-corrected *p*-value < 0.1, we identified a list of 60 transcripts in total, which we used to describe differences in the SARS-CoV-2 inoculated organotypic slices. (Figure 5B). These transcripts were annotated based on associated cell-type by comparison with the top cell-type enriched transcripts [31], and then were used for hierarchical clustering (Figure 5C). Differentially expressed transcripts at each time point were also functionally annotated using Enrichr (Figure 5D–I). Of note, we found that samples from tissue infected with SARS-CoV-2 collected at later time points, 5 and 14 DPI formed a separate cluster with a gene expression pattern that appeared distinct from the rest of the samples. Notably, there was a cluster of genes that were all annotated as microglia-associated that were decreased at these time points. Among these were well-characterised markers of microglia, including *Csf1r*, *Fcrls*, *Cx3cr1* and *P2ry12*. Consistent with this, many of the enriched pathways among decreased genes at these time points were related to regulation of macrophage differentiation, lysosomes and cytokine signalling (Figure 5G,I). There was also a cluster of transcripts that were strongly increased in abundance at 14- DPI, including *Ndrg2*, *Acan*, *Lox*, *Vegfa*, *Car9*, *Slc16a3* and the only neuron-associated transcripts were *Cplx2* and *Crym*. Altogether, the few transcriptional differences observed in SARS-CoV-2 treated tissues were consistent with limited infection.

## 4. Discussion

We show that SARS-CoV-2 was not neuroinvasive following infection in an established hamster model despite evidence of mild microglial activation throughout the brain that was most evident in the hippocampal region. Recent studies available as pre-prints have also reported similar levels of microglial activation as indicated by increased levels of staining using antibodies to microglial proteins and changes in morphology [35,36]. Additionally, transcriptional changes indicative of mild neuroinflammation have been observed in the brains of hamsters infected with SARS-CoV-2 that differed depending on the variant [36]. Microglial activation in the brainstem and hippocampus of elderly patients has been suggested to be the cause of “COVID-19 encephalopathic syndrome” in the elderly [37]. We used a hamster organotypic culture that preserves much of the intercellular complexity of brain tissue while circumventing the BBB to determine whether cells within the brain are able to support infection by SARS-CoV-2. We demonstrate that hamster cerebellar sections are relatively resistant to infection. By staining for the nucleocapsid protein and viral RNA using in situ hybridization we observed a small number of glial cells that were permissive for infection by SARS-CoV-2. We also noted the presence of live virus in the culture media that waned over time, suggesting non-productive infection of a small number of cells. Notably, all infected cells had morphologies closely resembling that of glia, and we saw no evidence of neuronal infection.

A major limitation of the cerebellar slice cultures are the changes that occur after they cultured. We accounted for this by comparing the transcriptional profiles over time in vitro (see Supplementary Figure S5). Many of the altered transcripts over time were categorized as enriched in vascular endothelial cells and were related to angiogenesis and cell-to-cell junctions, consistent with the vasculature being disrupted upon culturing. Of note, many of the transcripts with increased abundance were enriched in ontologies related to type I interferon signalling and the defence response to virus, potentially making these cultured brain sections less favourable for viral replication compared to the tissue in vivo.

Our finding of SARS-CoV-2 infection in a small number of GFAP^+^ astrocytes is consistent with other studies that have shown human astrocytes to be susceptible to SARS-CoV-2 [38,39,40]. We observed strong correlation between GFAP and nucleocapsid staining, although we did not always observe co-localization to individual cell processes. GFAP is an intermediate filament protein that is found in the major cellular projections of astrocytes but not the fine processes. The staining pattern we saw could reflect virus being transported along astrocytic processes that do not contain intermediate filaments. A second explanation would be that virus was infecting a different cell type, such as ACE2 expressing endothelial cells, that are tightly associate with astroglia. Therefore, further studies that closely examine SARS-CoV-2 localization in astrocytic processes and/or brain microvasculature may help shed further light on SARS-CoV-2 neuroinvasiveness. We also observed rare infection of IBA^+^ microglia; microglial infection was also recently reported by Jeong et al. in a study that is available as a pre-print [41].

Contrary to some other studies that have modelled SARS-CoV-2 infection in cultured human brain organoids [42,43,44] and a recent study that also used hamster brain slices [45], we failed to find evidence of neuronal infection in organotypic brain sections. Cultured organoids differ from mature brain tissue and organotypic sections in several ways, most markedly in the relative immaturity of cells and the lack of immune cells and vasculature. It is possible that receptors expressed by immature neurons not present in our cultured slices support infection with SARS-CoV-2 explain these differences. Viral infection was reported in brainstem organotypic slices by Ferren et al. [45] specifically in granular neuronal subtypes (Golgi type 1). The biological differences in subpopulations of cells within the slices and the culture conditions may explain the discrepancies between our observations and this study.

The major receptors used by SARS-CoV-2, such as ACE2 and the host-protease TMPRSS2, are necessary for entry to cells and negligibly expressed in brain tissue. Studies using mouse models that overexpress ACE2 throughout the body do show severe neurological signs and brain pathology, which suggests that the presence of appropriate receptors dictates tissue tropism [46]. Once virus enters the brain of these transgenic mice, multiple types of cells support viral replication, although the cortex and hippocampus were found to be more permissive than cells of the dentate gyrus and cerebellum [14]. We also examined the level of expression of SARS-CoV-2 receptors in our transcriptional data from the hamster cerebellar organotypic slice cultures. Although the level of expression of *Ace2* and *Dpp4* were negligible, the putative receptors *Nrp1* and particularly *Bsg* were abundantly expressed [47,48] (Supplementary Figure S6). We also looked at the expression of these genes in human and mouse brain single cell sequencing databases publicly available at the Allen Brain Atlas resource (https://atlas.brain-map.org/, access on 26 April 2022), but did not see any evidence of their expression within glial cells that would correlate with our findings. Therefore, the low abundance of ACE2 in the cerebellar sections may explain the non-productive infection observed here, but the expression of *Nrp1* and *Bsg* does not fully account for the permissiveness of glial cells.

ACE2 is highly expressed by brain endothelial cells, and possible inflammation and BBB disruption during SARS-CoV-2 infection may provide an entry point to the brain. Some have suggested other routes of neuroinvasion such as the olfactory nerves or retrograde transport via the vagus nerve; however, most recent reports suggest that such transport is likely to be very rare and evidence of viral replication in olfactory receptor neurons is lacking [49,50,51]. Autopsy studies have failed to provide convincing evidence of viral replication in the brains of diseased patients. Of note, a recent pre-print manuscript by Florent et al. [52] provides some evidence that virus can reach the hypothalamus where it specifically infects gonadotropin-releasing hormone secreting neurons. These neurons constitute only a few thousand cells, but it is suggested that their infection could result in the altered reproductive function that has been reported by some, including lower levels of testosterone [53,54]. Another pre-print manuscript [38] reports that brain tissue samples from five patients that died of COVID-19 showed neurologic signs such as cognitive dysfunction, exhibited foci of viral infection and replication in astrocytes.

We used RNAseq to explore any molecular changes that might be associated with the observed infection of glial cells with SARS-CoV-2. Given the sparsity of infected cells, we were only able to identify a small number of genes that were differentially expressed, and therefore these results should be interpreted with caution. Nonetheless, the most striking pathway that was induced over time was the HIF1a pathway that regulates expression of the genes VEGF, ADM and GPI (Figure 5C). Moutal et al. [55] demonstrated that the S1 domain of the SARS-CoV-2 spike protein interacts with the extracellular domain of NRP1, inhibits VEGF-A mediated spontaneous firing of neurons in dorsal root ganglia and identified a novel VEGF-A/NRP1 pathway that drives nociception. Thus, transcriptional activation of the VEGF-A pathway is consistent with the receptor NRP1 being expressed within the brain slices. The expression of *Vegfa* expression is increased in patients with COVID-19 and is associated with disease severity [56,57,58]. The drug bevacizumab, a humanized monoclonal antibody that binds VEGF, was found to be beneficial in the treatment of patients with severe COVID-19 [59]. VEGF is a potent vascular permeability factor that induces vascular leakiness and permeability of the endothelial cells in the lungs of infected patients and potentially of the BBB; perhaps contributing to the neurological manifestations of COVID-19. Similarly, another gene involved in BBB integrity, N-myc downstream-regulated gene 2 (*Ndrg2*), that is principally expressed in astrocytes had increased expression at 14 DPI [60]. In silico simulations predict binding of SARS-CoV-2 spike protein to *Ndrg2* and further suggests the potential for the triggering of inflammation at the BBB [61].

## 5. Conclusions

In summary, we find that a small population of glial cells in hamster cerebellar sections were permissive for SARS-CoV-2, although infection was non-productive even in the absence of a BBB. We speculate that in severe disease, a disrupted BBB could lead to localized infection of glia and may explain the findings of a small number of studies in which viral proteins and RNA have been detected in brain tissue of patients and animals. However, the paucity of SARS-CoV-2 infection observed here aligns more closely with other reports that failed to identify evidence of neuroinvasion. Evidence from experimental models remains inconclusive, and careful examination of clinical cases will undoubtedly be required to shed further light on the troubling long-term neurological manifestations associated with COVID-19.

## Data Availability

The raw data generated in RNA-seq study were submitted to the SRA database https://www.ncbi.nlm.nih.gov/sra) (accessed on 29 April 2022) under accession number GSE201921 (release date: 6 May 2022).

## Supplementary Materials

The following supporting information can be downloaded at: https://www.mdpi.com/article/10.3390/v14061218/s1, Figure S1: Absence of SARS-CoV-2 nucleocapsid protein staining in the hippocampus and thalamus of infected hamsters, Figure S2: SARS-CoV-2 infected glial cells in hamster brain cerebellar slice culture, Figure S3: In situ hybridization for SARS-CoV-2 RNA colocalizes with GFAP, Figure S4: SARS-CoV-2 does not infect neuronal cells in an organotypic cerebellar hamster brain slice cultures, Figure S5: Transcriptional changes in cerebellar slice cultures infected with SARS-CoV-2 over time, Figure S6: SARS-CoV-2 receptor expression in hamster cerebellar slices, Table S1: List of Antibodies used. Refs. [62,63] were cited in supplementary material.
