# Supplementary material for: Non-Productive Infection of Glial Cells with SARS-CoV-2 in Hamster Organotypic Cerebellar Slice Cultures
Source: Viruses. 2022 Jun 3;14(6):1218. doi: 10.3390/v14061218 (PMC9227386; doi:10.3390/v14061218)
Supplement: Supplementary file 1 [file viruses-14-01218-s001.zip › viruses-1732151-supplementary.pdf]

Supplemental data:

Table S1: List of Antibodies used.

Primary antibodies					
**Species**	**Epitope**	**Company**	**ID#**	**Dilution (1:xxxx)**	**Assay**
Rabbit	SARS-CoV2- Nucleocapsid	Sino Biological	40143-R019	2000	IIF
Mouse	β3-Tubulin	Abcam	ab78078	5000	IIF
Mouse	Iba1	Invitrogen	GT10312	1000	IIF
Chicken	Gfap	Abcam	ab4674	2000	IIF
Rabbit	NeuN	Abcam	ab177487	1:2000	IIF
**Secondary antibodies**					
Goat	Rabbit IgG- AlexaFluor 488	Invitrogen	A11034	1000	IIF
Goat	Mouse IgG- Cy3	Abcam	ab97035	750	IIF
Goat	Chicken IgY- AlexaFlour 647	Invitrogen	A32933	1000	IIF
**Opal Dyes**					
	Opal 520	Akoya Biosciences	FP1487001KT	2000	ISH
	Opal 570		FP1488001KT	1200	ISH
	Opal 690		FP1497001KT	1500	ISH

<u>Figure S1: Absence of SARS-CoV-2 nucleocapsid protein staining in the hippocampus and thalamus of SARS-CoV-2 infected hamsters.</u>

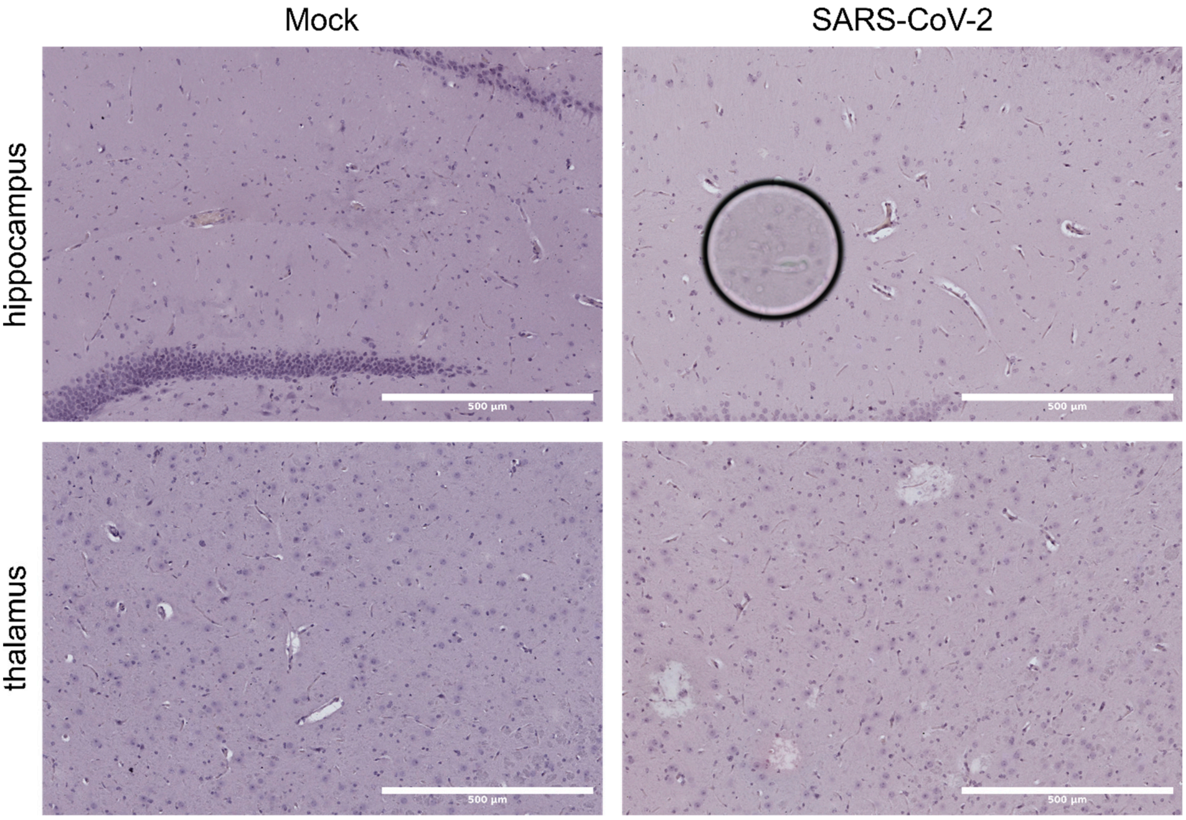

IHC images showing staining for SARS-CoV-2 nucleocapsid protein in the hippocampus and thalamus of brain sections taken from hamsters that were infected with SARS-CoV-2. Scale bar indicates 500 µm.

<u>Figure S2: SARS-CoV-2 shows mild infection of cells in hamster brain cerebellar slice culture.</u>

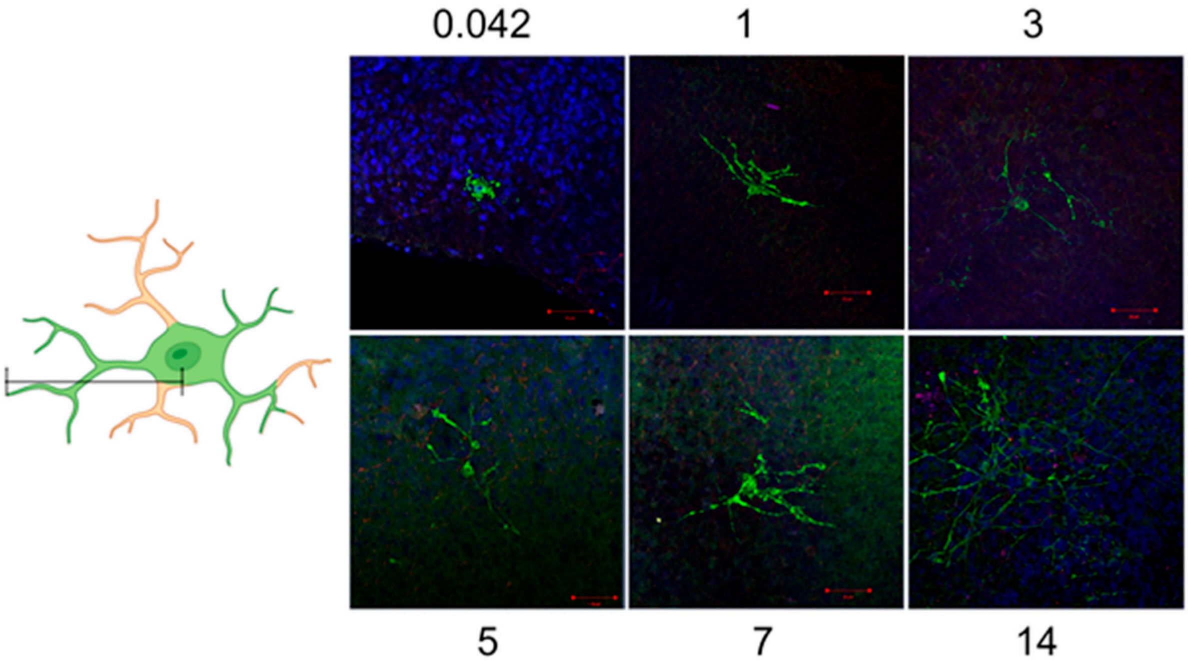

Representative pictorial depiction of measurement of maximum signal intensity of an infected glial cell (left); and collage of images (right) from infected hamster brain cerebellar slices over 14 DPI. Viral nucleocapsid was visualized by staining with antibodies and detected as a green fluorescent signal. The maximal distance of continuous signal from the center of the cell was calculated as described in methods.

Figure S3: In situ hybridization identifies overlapping Gfap and SARS-CoV-2 RNA.

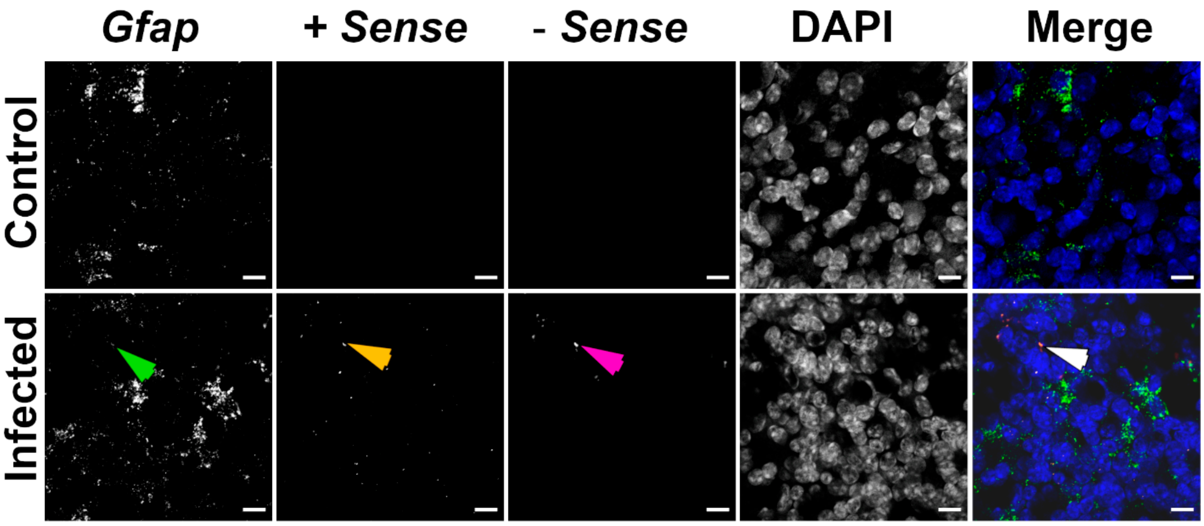

Representative images of hamster brain cerebellar slices labelled to visualize astrocytic (*Gfap*; green arrowhead) and SARS-CoV-2 markers; +Sense (yellow arrowhead), –Sense (magenta arrowhead) viral RNA strands indicating areas of active infection and replication respectively by RNA ISH. Nuclei were counterstained with DAPI/ blue; merge depicts a composite image. Scale bar = 10µm.

Figure S4: SARS- CoV-2 does not infect neuronal cells in an organotypic cerebellar hamster brain slice cultures.

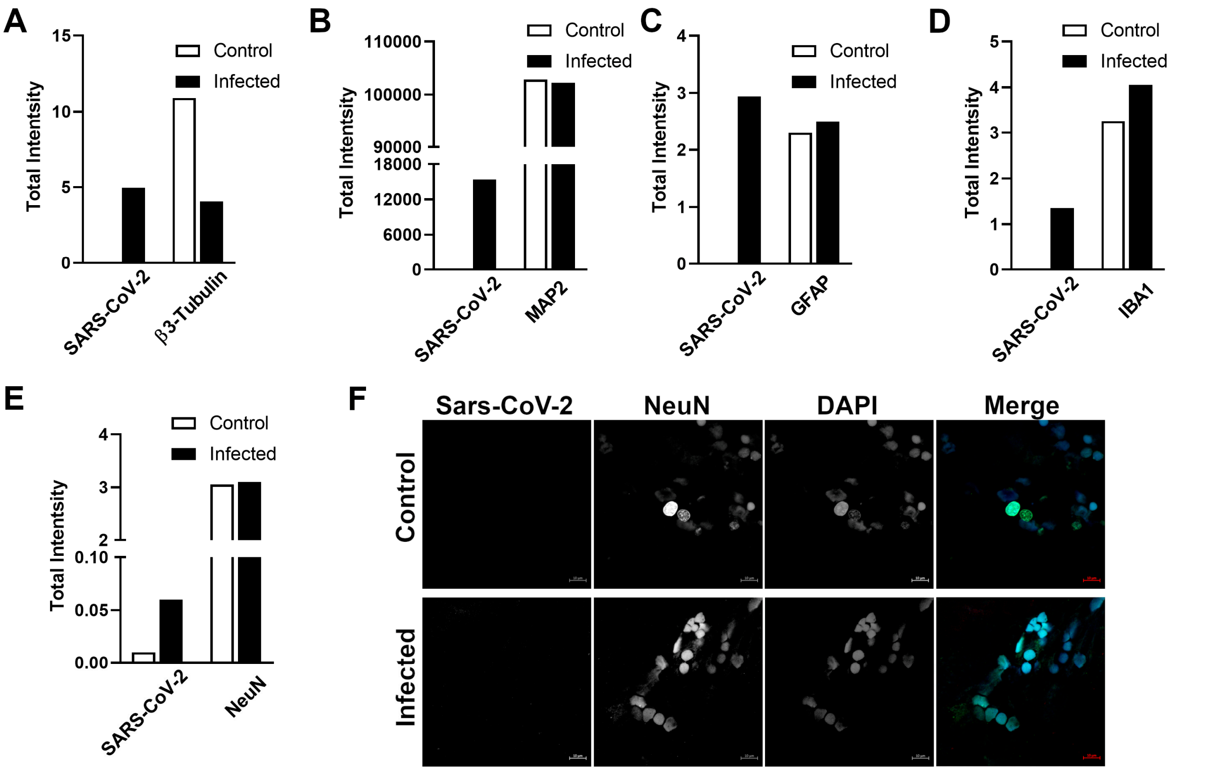

A-E) Corrected total fluoresecene intensity for SARS-CoV-2 and markers for neurons (β3-Tubulin; A, Map2; B, NeuN; E) and glial cells (Gfap; C, Iba1; D) in hamster organotypic cerebellar slice cultures. F) Indirect immunofluorescence images of organotypic cerebellar slice cultures stained for SARS-CoV-2 (+ sense; ISH) and neuronal marker NeuN. DAPI was used to counterstain nucleus and merge represents a composite image. Bar=10µm.

<u>Figure S5: Transcriptional changes in cerebellar slices after 5- and 14-days in vitro.</u>

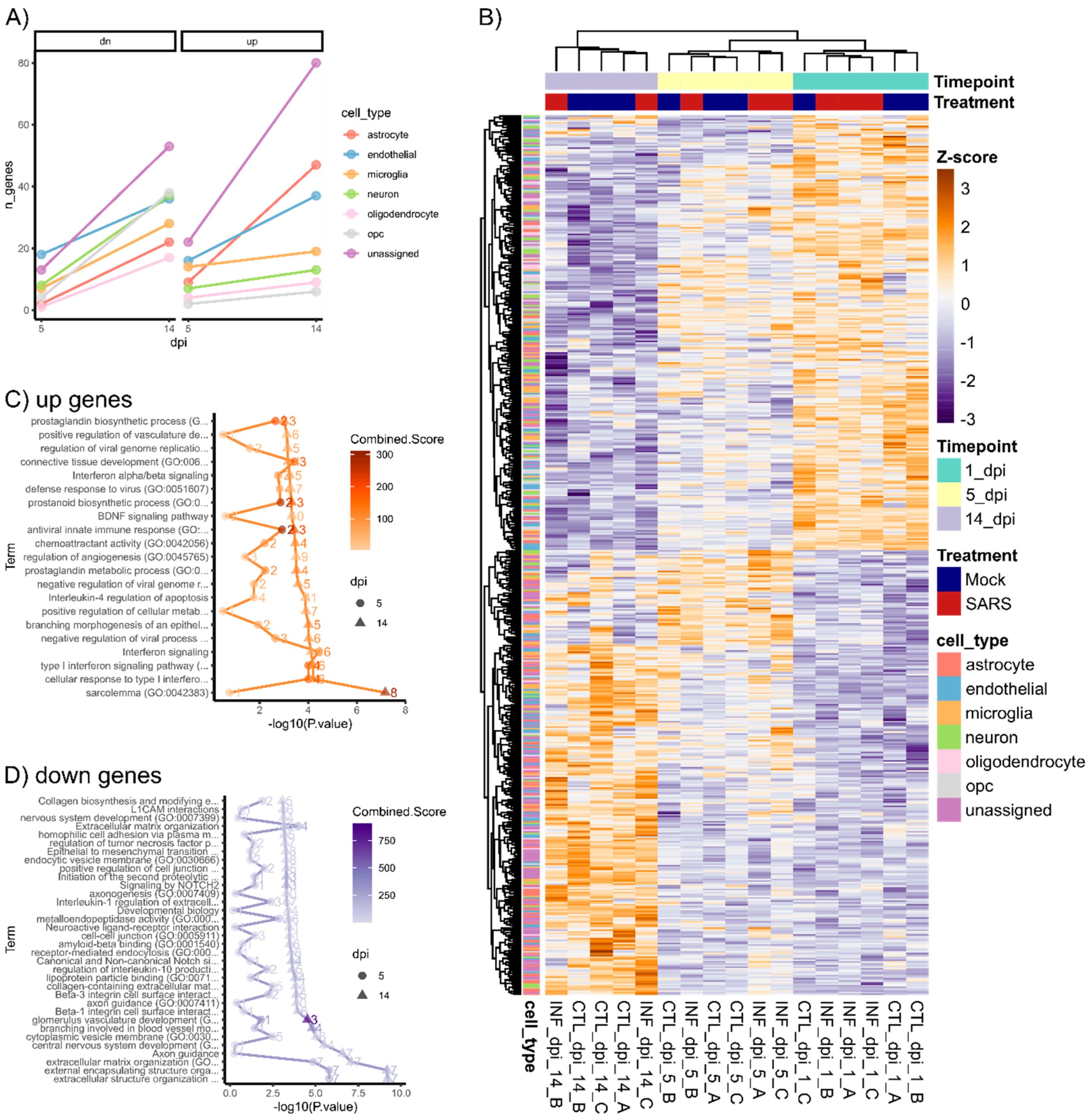

(A) Number of up- and dn-genes identified as differentially expressed in cerebellar slices at day-5 and day-14 compared to day-1. Genes were categorized into neural cell types and used for (B) hierarchical clustering. A pattern of genes that either progressively increase in abundance, or decrease, can be seen. Enrichr was used to identify enriched gene sets among (C) increased and (D) decreased genes at day-5 and day-14 compared to day-1 *in vitro*.

Figure S6: Levels of putative SARS-CoV-2 receptors.

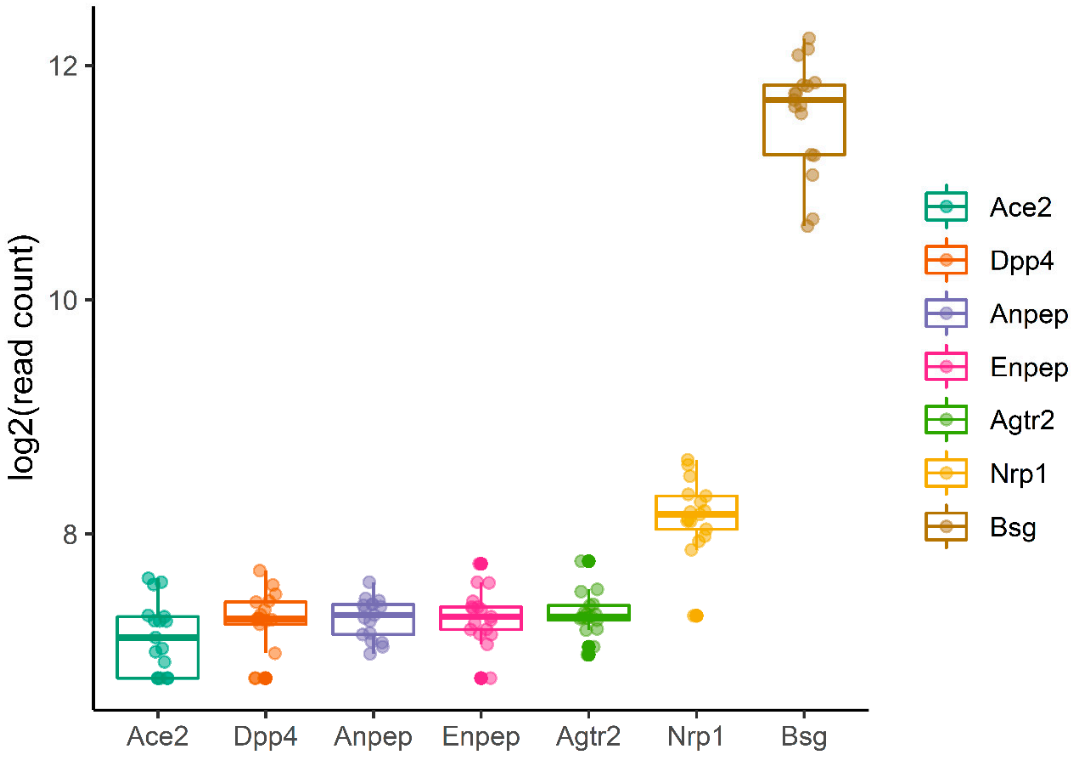

RNAseq raw read counts mapping to SARS-CoV-2 putative receptor transcripts were normalized and abundance was calculated as log2 transformed read counts using DESeq2.

Supplementary Methods:

<u>*Quantitation of signal intensities from fluorescence images:*</u> Fluorescent images were acquired and saved as TIFF files as described earlier with constant exposure times between control and infected samples. Uncompressed TIFF image files were opened in Fiji [1] to calculate corrected intensity of fluorescent signals (represented as total intensity) of SARS-CoV-2 signal and other markers as described elsewhere [2]. Briefly, 3-6 individual areas of signal (regions of interest; ROI) was selected using a polygonal selection tool for all markers and at least 3 such ROIs from each image with no signal was used to determine background fluorescence. Using measure tool in Fiji Area, mean and raw intensities were collected for each ROI. A mean of raw intensities of background was calculated. Values were exported into Excel® and the corrected total fluorescence intensity (TI) was calculated using the formula TI = [Raw intensity – (area × mean intensity of background). Values were imported into GraphPad Prsim ™ version 9.3.1., and bar diagrams were generated.

Supplementary References:

1. Schindelin, J., Arganda-Carreras, I., Frise, E., Kaynig, V., Longair, M., Pietzsch, T., … Cardona, A. (2012). Fiji: an open-source platform for biological-image analysis. *Nature Methods*, *9*(7), 676–682. doi:10.1038/nmeth.2019

2. Martin Fitzpatrick. (2014). https://theolb.readthedocs.io/en/latest/imaging/measuring-cell-fluorescence-using-imagej.html
